# S-layer fusion protein as a tool functionalizing emulsomes and CurcuEmulsomes for antibody binding and targeting

**DOI:** 10.1016/j.colsurfb.2015.01.055

**Published:** 2015-04-01

**Authors:** Mehmet H. Ucisik, Seta Küpcü, Andreas Breitwieser, Nicola Gelbmann, Bernhard Schuster, Uwe B. Sleytr

**Affiliations:** a Institute for Synthetic Bioarchitectures, Department of Nanobiotechnology, University of Natural Resources and Life Sciences (BOKU) Vienna, Muthgasse 11, 1190 Vienna, Austria; b Department of Biomedical Engineering, School of Engineering and Natural Sciences, Istanbul Medipol University, Ekinciler Cad. No. 19 Kavacık Kavşağı, Beykoz 34810, Istanbul, Turkey; c Institute for Biophysics, Department of Nanobiotechnology, University of Natural Resources and Life Sciences (BOKU) Vienna, Muthgasse 11, 1190 Vienna, Austria; d Octapharma GmbH, Oberlaaer Straße 235, 1100 Vienna, Austria

**Keywords:** Emulsomes, Curcumin, S-layer (fusion) proteins, Immunoglobulin G (IgG) Targeting, Active drug delivery

## Abstract

Selective targeting of tumor cells by nanoparticle-based drug delivery systems is highly desirable because it maximizes the drug concentration at the desired target while simultaneously protecting the surrounding healthy tissues. Here, we show a design for smart nanocarriers based on a biomimetic approach that utilizes the building principle of virus envelope structures. Emulsomes and CurcuEmulsomes comprising a tripalmitin solid core surrounded by phospholipid layers are modified by S-layer proteins that self-assemble into a two-dimensional array to form a surface layer. One significant advantage of this nanoformulation is that it increases the solubility of the lipophilic anti-cancer agent curcumin in the CurcuEmulsomes by a factor of 2700. In order to make the emulsomes specific for IgG, the S-layer protein is fused with two protein G domains. This S-layer fusion protein preserves its recrystallization characteristics, forming an ordered surface layer (square lattice with 13 nm unit-by-unit distance). The GG domains are presented in a predicted orientation and exhibit a selective binding affinity for IgG.

## Highlights

• IgG targeting by the genetically engineered S-layer fusion protein rSbpA-GG.
• rSbpA-GG assembles to a uniform square monomolecular lattice on CurcuEmulsomes.
• The two protein G domains of each rSbpA-GG on CurcuEmulsomes are freely accessible.
• High affinity for IgG underlines the potential for targeted delivery of curcumin.
• This approach will allow the generation of tailored multifunctional surfaces on DDS.

## Graphical abstract

**Figure:**
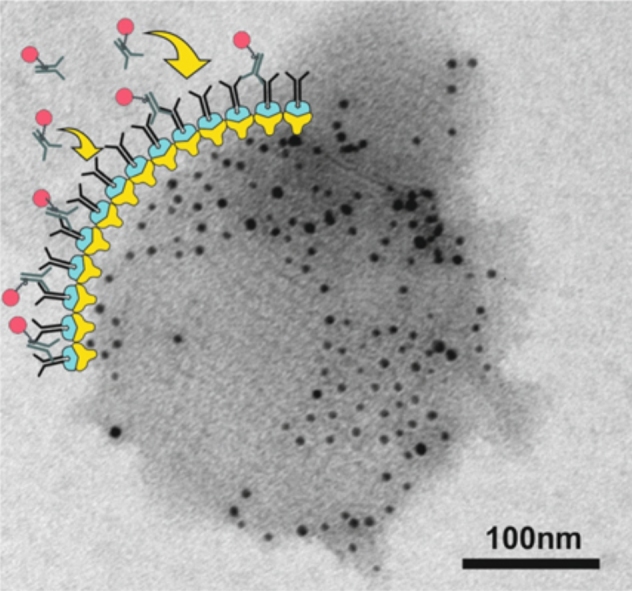

## 1 Introduction

Many studies provide evidence that the use of nanoparticle-mediated targeted drug delivery systems (DDS) minimizes drug degradation and inactivation upon administration while increasing drug bioavailability and the fraction of drug delivered in the pathological area [1–3]. Active targeting implies tailor-made surface modifications of nanoparticles with ligands that bind to specific receptors expressed on target cells.

In nature, viruses are extremely effective at targeting cells and delivering genomes into their host. One remarkable property of viruses is a regular and well-defined proteinaceous structure called a capsid [4]. This feature can also be observed on many bacteria and most archaea, which carry a crystalline cell surface (S-) layer as the outermost envelope structure [5,6]. Composed of a single protein or glycoprotein species [7], S-layer lattices can be employed to act as an anti-fouling layer [8] and to decrease the cytotoxicity of blank nanocarriers such as polymeric capsules [9] and emulsomes [10], thereby improving the bioavailability of enclosed drugs.

Members of the immunoglobulin superfamily are among the most common selected targets for directing drugs to cells of interest, particularly inflammatory and cancer cells [11]. Binding to immunoglobulin G (IgG) is known to be important for the opsonization process, which can further activate the classical component pathway presenting particulates or colloidal carriers to phagocytes [12]. It has also been reported that IgG secreted by human cancers promotes growth and survival of tumor cells [13]. Hence, IgG may serve as a potential therapeutic target in cancer therapy.

Lipid-based nanocarrier systems are attractive because of their biocompatibility, biodegradability and their ability to entrap hydrophilic and hydrophobic drugs [14]. Emulsomes are a form of lipid-based nanocarrier with an internal solid fat core surrounded by a phospholipid multilayer [15–17]. They offer both high loading capacity for hydrophobic substances [18] and controlled drug release [16,19]. Moreover, the phospholipid surface can be adapted to various surface modifications already established for liposomes to control the targeting or enhance circulation residence time [20–22]. The surface of emulsomes can also be coated and modified with S-layer proteins to bestow the nanocarriers with the characteristic features of the S-layer lattice [10].

Mimicking the building principle of virus envelopes, this study introduces an emulsome-based targeted DDS for the anti-cancer agent curcumin. The medical use of this natural polyphenol is currently limited due to its extremely low water solubility. The solubility of curcumin, however, can be enhanced via encapsulation in emulsomes, which produces nanocarriers that have been named CurcuEmulsomes [19]. CurcuEmulsomes not only facilitate the delivery of curcumin to the human liver carcinoma cell HepG2 in vitro, but also prolong the biological effect by triggering controlled drug release inside the cell [19].

The present study investigates how S-layer fusion technology can be applied to emulsomes and CurcuEmulsomes to promote targeted delivery to IgG. For this purpose, the fusion protein rSbpA-GG was designed with two protein G domains fused to the S-layer protein SbpA from *Lysinibacillus sphaericus* CCM 2177. The present study involves the genetic design and expression of rSbpA-GG and explores its self-assembly and IgG-binding characteristics on emulsomes. This study will contribute to the utilization of emulsomes for delivery of lipophilic therapeutics in nanomedicine.

## 2 Materials and methods

### 2.1 Materials

Curcumin, glyceryl tripalmitate (tripalmitin, purity ≥99%), 1,2-dipalmitoyl-rac-glycero-3-phosphocholine (DPPC, ∼99%), glutaraldehyde solution (50%), human IgG-Reagent Grade, anti-human IgG (γ-chain specific)-gold antibody, Sudan III and FITC were purchased from Sigma–Aldrich GmbH, Germany. Hexadecylamine (HDA, ≥99%), uranyl acetate dehydrate (≥98%) and chloroform (≥99.8%) were obtained from Fluka Chemika, Switzerland and Germany, respectively. Cholesterol (>%98) was purchased from Avanti Polar Lipids, USA. Dimethyl sulfoxide (DMSO) was purchased from Riedel-de Haën (Sigma–Aldrich, Germany) and isopropyl-β-d-thiogalactoside (IPTG) was purchased from Gerbu (Gaiberg, Germany). All chemicals were used as received without further purification.

### 2.2 Cloning of the recombinant plasmid pET28a/*rsbpA*_31-1068_/gg

All PCRs were performed as described in Jarosch et al. [23]. The two GG domains were amplified by PCR from plasmid SPG1 (Genart, Regensburg, Germany) containing two copies of the gene sequence encoding the G domain with a glycine/glycine linker in between as well as the restriction sites BamHI (upstream) and XhoI (downstream). The DNA sequence encoding the two G domains was ligated into the two corresponding restriction sites of plasmid pET28a containing the gene *rsbpA*_31-1068_ encoding S-layer protein rSbpA_31-1068_. Digestion of DNA with restriction endonucleases, separation of DNA fragments by agarose gel electrophoresis, ligation of DNA fragments and transformation procedures were performed as previously described [24].

### 2.3 Heterologous expression of the fusion protein rSbpA-GG

The construct pET28a–*rsbpA*_31-1068_/gg was cloned in *Escherichia coli* TG1 and heterologously expressed in *E. coli* BL21 (DE3) Star as previously described [24]. The plasmid stability test was performed as described previously [23]. Expression of the chimeric gene encoding rSbpA-GG was induced by the addition of IPTG to a final concentration of 1 mM at an attenuance (*D*_600_) of 0.6–0.8. Expression was carried out at 37 °C for 4 h. Samples (2 mL) were taken before and at 1, 2 and 4 h after induction of gene expression and run on SDS-PAGE. SDS-PAGE was performed as described previously [23].

### 2.4 Isolation of S-layer fusion protein SbpA-GG

After 4 h of expression, isolation of rSbpA-GG from *E. coli* BL21(DE3) Star cells was performed using Bacterial Protein Extraction Reagent (B-PER) following a modified protocol. The B-PER was purchased from Pierce (Rockford, IL, USA). Biomass pellets (2 g) were initially resuspended in 20 mL of B-PER. After incubation for 10 min at room temperature the solution was centrifuged (20,000 × *g*, 15 min, 4 °C). Subsequently, the pellet was resuspended in 20 mL of B-PER containing and 4 mg of lysozyme (Sigma, Munich, Germany) and incubated for 5 min at room temperature. 20 μL of 1 mg mL^−1^ DNAseI solution (Roche, Basel, Switzerland) and 1 mL of a 0.1 M MgSO_4_·7H_2_O solution were added and the suspension was incubated for 30 min at room temperature. Subsequently, 100 mL of B-PER (diluted 1:10 in distilled water) was added and the solution was centrifuged (20,000 × *g*, 20 min, 4 °C). After two washing steps with 30 mL of diluted B-PER and one washing step with 30 mL of 50 mM Tris/HCl buffer (pH 7.2) with 1 mM dithiothreitol (DTT), the suspension was centrifuged (20,000 × *g*, 20 min, 4 °C) and the obtained pellet was resuspended in 3.5 mL of 4 M guanidine hydrochloride (GHCl) dissolved in 50 mM Tris/HCl buffer (pH 7.2) and stirred for 30 min at room temperature. The suspension was diluted to a final GHCl concentration of 2 M. To remove membrane fragments, the suspension was centrifuged (36,000 × *g*, 30 min, 4 °C), the supernatant was filtered through a filter membrane with pore size of 0.45 μm (Minisart RC 25) and the filtrate was subjected to GPC (gel-permeation chromatography) using a Superdex 200 column (Amersham Biosciences, Little Chalfont, Bucks, UK) equilibrated in degassed 50 mM Tris/HCl buffer (pH 7.2) with 150 mM NaCl, for separation. Fractions containing the fusion protein rSbpA-GG were pooled, dialysed against Milli-Q water for 18 h at 4 °C, lyophilized and stored at −20 °C. For detection of the GG portion, the fusion protein rSbpA-GG was subjected to SDS-PAGE.

### 2.5 Preparation of rSbpA-GG ELISA plates

Lyophilized rSbpA-GG was dissolved in 5 M GHCl/Tris buffer (pH 7.2) and dialysed against 3 L Milli-Q water at RT for 3 h (BioMol: Dialysis membrane type 8; cut-off 12–16 kDa). Water was changed three times: after 30 min, 60 min and then after 90 min. Subsequently the solution was centrifuged at 14,000 rpm for 5 min and the protein concentration of the supernatant was determined using UV 280 nm measurements and adjusted to 1 mg mL^−1^. The solution was diluted with crystallization buffer (0.5 mM Tris, 10 mM CaCl_2_, pH 9.5) to a final concentration of 100 μg mL^−1^ protein. From this solution 110 μL was transferred to each well of an ELISA plate (Microlon 200; GREINER, medium capacity). Crystallization was performed at 4 °C overnight. Unbound S-layer protein was washed away with crystallization buffer. Subsequently, the S-layer protein was stabilized and blocked by an incubation step with 250 μL StabilGuard (SurModics; 1:1 PBS/Triton X 100) per well at RT for 4 h. The liquid was removed and the plates were dried at 37 °C overnight.

### 2.6 Estimation of IgG binding affinity of rSbpA-GG by ELISA

The rSbpA-GG plates were incubated with peroxidase-labeled anti-human IgG developed in goat (=anti-human IgG POX, Sigma A0293 F_ab_ specific developed in goat; 1:5000 in blocking buffer) at RT for 30 min. After 3 washing steps with 250 μL PBS/Triton X-100, bound IgG POX was detected using 3,3′,5,5′-tetramethylbenzidine dihydrochloride (TMB; Sigma, T-3405) as substrate; 200 μL TMB solution was added per well and the color development was stopped by addition of 50 μL 2 M H_2_SO_4_. Subsequently, the yellow color of the samples was read at 450 nm (reference filter 630 nm) with an ELISA Reader. As a control, an identical assay was performed with rSbpA and the S-layer fusion protein rSbpA-ZZ comprising protein A [24].

### 2.7 Preparation of emulsomes and CurcuEmulsomes

Emulsomes were prepared as described previously [10]. CurcuEmulsomes with two different curcumin concentrations were prepared as described in Ucisik et al. [19]. Accordingly, one preparation has prepared as before with the curcumin–tripalmitin weight ratio of 2:5 [19], whereas the other with a weight ratio of 1:10. The DPPC, cholesterol and HDA molar ratio was as before 10:5:4. The suspension was filtered at 66 °C through polycarbonate filters (three passes through 800 nm pore filters, followed by 2 passes through 400 nm pore filters; filters from Nucleopore Track Etch Membrane, Whatman, UK). The filtrate was placed immediately on ice for a 10 min period, followed by centrifugation at 13,200 rpm (16,100 × *g*) for 10 min to spin down unincorporated curcumin. The CurcuEmulsome suspension, i.e., the supernatant, was stored at 4 °C.

### 2.8 Recrystallization of wtSbpA and rSbpA-GG on emulsomes

1 mg lyophilized S-layer protein was dissolved in 1 mL 5 M GHCl 50 mM Tris/HCl buffer (pH 7.2). The solutions were dialysed against distilled water at least 24 h at 20 °C. For recrystallization of the S-layer protein on emulsomes, the S-layer protein solution was mixed with the emulsome suspension and diluted with Milli-Q water to achieve final protein and DPPC concentrations of 300 μg mL^−1^ and 150 μg mL^−1^, respectively. Recrystallization of the S-layer protein was carried out for 3 h at room temperature in a test tube rotator (REAX2, Heidolph, Germany) with a rotation speed of 32–36 rpm. Excess non-assembled S-layer protein was removed by centrifugation at 14,100 × *g* for less than 1 min. The pellet containing the S-layer coated emulsomes was resuspended in Milli-Q water and stored at 4 °C until further analysis.

### 2.9 Affinity assay with human IgG (HIgG) and anti-human IgG gold conjugates (α-HIgG-Au)

Reagent grade HIgG was dissolved in 10 mM PBS (pH 7.4) with a final antibody concentration of 500 μg mL^−1^. Emulsomes coated with rSbpA-GG were mixed with this solution in a 1:2 protein mass ratio, and the volume was adjusted with 10 mM PBS (pH 7.4) to achieve a final emulsome concentration of 150 μg DPPC mL^−1^ (i.e., final HIgG concentration = 300 μg mL^−1^). The obtained mixture was incubated for 2–2.5 h at room temperature in the test tube rotator with a rotation speed of 32–36 rpm. The sample was then centrifuged for 2 min. The supernatant was discarded, the pellet was resuspended in 10 mM PBS (pH 7.4) and the centrifugation was repeated. Again the supernatant was discarded and the pellet was dissolved in 50 μL 10 mM PBS (pH 7.4). The obtained product, corresponding to HIgG-immobilized rSbpA-GG coated emulsomes, was incubated in first 1:10 and then 1:5 diluted γ-chain specific α-HIgG-Au (G0786, Sigma–Aldrich, Germany) for 10 and 15 min, respectively, at 20 °C in the Eppendorf thermomixer (Eppendorf, Austria). Accordingly, 10 μL of rSbpA-GG + HIgG coated emulsome, corresponding to nearly 5 μg of DPPC, was mixed with 10 μL of 1:5 diluted α-HIgG-Au conjugate and incubated for 10 min. The solution was centrifuged 90 s at 14,100 × *g*, and the colorless supernatant was discarded. The incubation was repeated this time by directly applying 10 μL of 1:5 diluted α-HIgG-Au conjugate onto the pellet. The mixture was incubated 15 min at 20 °C in an Eppendorf thermomixer, after which it was centrifuged and the supernatant was removed. The faint red color of the supernatant indicated that free gold conjugates were present in the broth, implying that saturation should be reached. Pellets were dissolved with Milli-Q water or 10 mM PBS solution (pH 7.4). The final product was analyzed by TEM after negative staining as described in Ucisik et al. [10].

### 2.10 TEM

The shape, the integrity of the emulsomes, and the lattice of recrystallized S-layer proteins were analyzed with a FEI Tecnai G2 20 Transmission Electron Microscope (TEM) at 80 kV equipped with FEI Eagle 4k camera (FEI Europe, The Netherlands) after a negative stain preparation.

### 2.11 Quantification of curcumin by absorbance measurements

Curcumin concentration in samples was estimated as described previously [19]. Sample absorbance was measured at 430 nm using an Infinite F200 plate reader (TECAN, Austria).

### 2.12 Dynamic light scattering and zeta potential

Emulsomes in 1 mM KCl solution (pH 6.3) to a final DPPC concentration of 4 μg mL^−1^ were analyzed with a Zetasizer (Zetasizer Nano ZS, Malvern Instruments Ltd., UK) to determine the particle size distribution (dynamic light scattering; DLS) and zeta potential (Phase Analysis Light Scattering; M3 PALS). The zeta potential values were calculated from the electrophoretic mobility using the Smoluchowski model [10]. The conductivity of the buffer varied in the range of 0.16–0.18 mS cm^−1^ at each measurement.

## 3 Results

### 3.1 Heterologous expression of the fusion protein rSbpA-GG

**Fig. 1:**
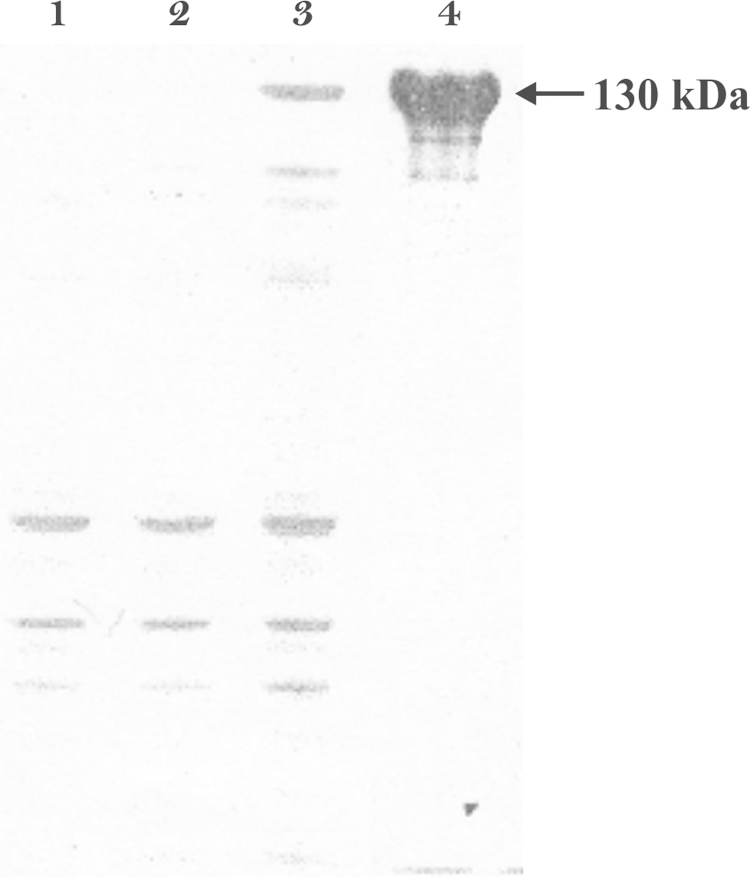
SDS/PAGE pattern of protein extract of *E. coli* BL21 (DE3) Star cells: (1) before induction; (2) 2 h after induction; (3) 4 h after induction; and (4) after purification.

The optimum expression of the fusion protein was found to be at 4 h after induction (Fig. 1, lane 3). SDS-PAGE of samples collected during the isolation procedure showed that rSbpA-GG had accumulated in the insoluble fraction of the lysed *E. coli* BL21 (DE3) Star host cells (data not shown). Following gel-permeation chromatography, SDS-PAGE analysis showed a single protein band with an apparent molecular mass of 130 kDa (Fig. 1, lane 4) which confirmed the purity of the protein. Membrane proteins are known to size anomalously on SDS-PAGE calibrated with conventional standards [25], explaining the high relative molecular mass value in comparison to the formula molecular weight of 116 kDa. The rSbpA-GG was expressed and purified with an overall yield of 55 mg protein out of 2 g wet biomass pellet.

### 3.2 Self-assembly properties of rSbpA-GG

Purified rSbpA-GG was recrystallized on poly-l-lysine (PLL) pre-coated copper grids and on a silicon wafer. As shown by TEM images of negatively stained preparations, rSbpA-GG reassembled into flat sheets, which clearly exhibited the square (p4) lattice structure of wild type (wt) SbpA (see Supplementary data Fig. 1A). The self-assembly characteristics of the rSbpA-GG were also verified on a planar silicon wafer via AFM studies (Supplementary data Fig. 1B).

### 3.3 Detection of IgG binding properties of rSbpA-GG

**Fig. 2:**
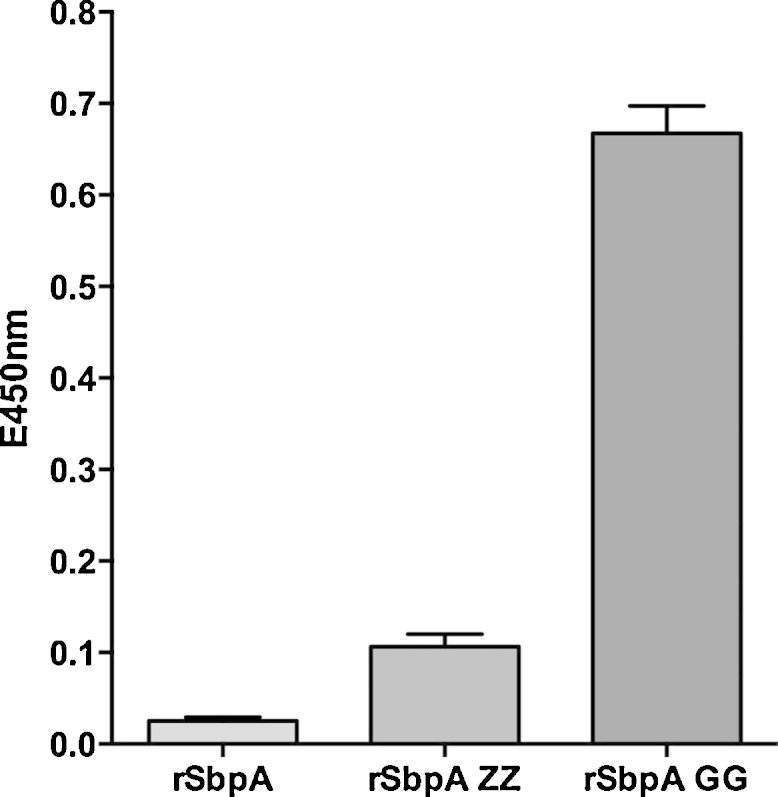
ELISA results showing binding affinity of HIgG POX (developed in goat) on reassembled (from left to right) rSbpA, rSbpA-ZZ and rSbpA-GG monolayers obtained by the ELISA. Developed yellow color was read at 450 nm on a microplate reader. The standard deviation (SD) is given as bars within the graph (*n* = 3).

ELISA plates coated with rSbpA-GG were incubated with peroxidase (POX) labeled HIgG (developed in goat). The color change after application of POX substrate confirmed the binding. For controls, parallel studies were performed with (i) recombinant (*r*) S-layer protein rSbpA lacking IgG binding domains, as well as (ii) previously engineered rSbpA-ZZ proteins comprising two protein A domains fused to the SbpA [24]. The goat IgG bound to ELISA plates coated with rSbpA-ZZ at a much lower level (Fig. 2), which is in accordance with the weak affinity of protein A for goat IgG as described in literature [26,27]. Plates coated with rSbpA exhibited as expected no significant binding of IgG. The results obtained clearly demonstrated that goat IgG was only bound to rSbpA-GG coated plates, confirming the species independent IgG affinity of protein G, and also the fact that protein G molecules genetically fused to the S-layer preserve their distinctive IgG binding characteristics.

### 3.4 Emulsomes

Emulsomes were characterized with respect to their intrastructure and physical characteristics as described in detail by Ucisik et al. [10]. Following the same methodology, the average diameter of emulsomes was found to be 297 ± 28 nm and zeta potential was 32.4 ± 5.9 mV. The confidence intervals represent the variation of average values of different formulations.

### 3.5 CurcuEmulsomes

In the present study, the curcumin concentration in the suspension was either 30 or 110 μg mL^−1^, with the curcumin being concentrated inside the solid fat core of the emulsomes. Unless otherwise specified, all results described in this work refer to CurcuEmulsome suspensions with 30 μg mL^−1^ curcumin. This curcumin concentration in the suspension corresponds to a 2700-fold increase over the 11 ng mL^−1^ maximum solubility of curcumin in water [28]. The incorporation of curcumin did not influence either the size or the zeta potential characteristics of the emulsomes significantly. CurcuEmulsomes are spherical in shape with an average diameter of 291 ± 48 nm and have an average zeta potential of 29.8 ± 2.1 mV. The confidence intervals represent the variation of average values of different formulations.

### 3.6 Recrystallization of rSbpA-GG on emulsomes and CurcuEmulsomes

**Fig. 3:**
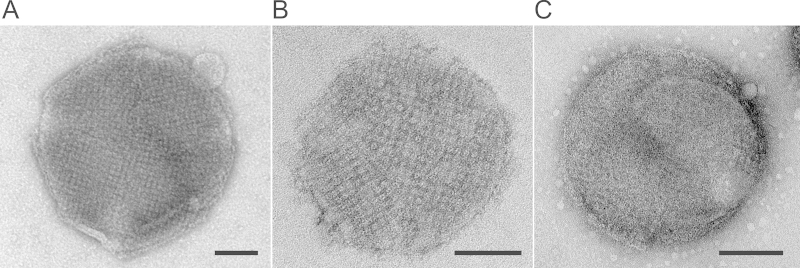
TEM images of (A) an emulsome; (B) a CurcuEmulsome completely covered by the S-layer fusion protein rSbpA-GG; (C) an emulsome coated with wtSbpA. Bar sizes correspond to 100 nm.

Upon recrystallization, rSbpA-GG coated the entire surface of both emulsomes and CurcuEmulsomes, assembling into the characteristic square lattice symmetry as evidenced by TEM (Fig. 3). It is important to emphasize that rSbpA-GG (Fig. 3A and B) displays the same lattice symmetry as wtSbpA (Fig. 3C), i.e., unit-by-unit distance of 13.1 nm and base angle *γ* = 90° [29].

The recrystallization of the S-layer protein altered the zeta potential of the nanoformulations. Hexadecylamine present in the phospholipid layer confers a net positive charge (Table 1), which upon coating with rSbpA-GG became negative: the zeta potentials were found to be −19.5 ± 3.7 mV and −22.7 ± 3.7 mV for coated emulsomes and CurcuEmulsomes, respectively. These values were comparable to the zeta potential of emulsomes coated with wtSbpA, which was −18.7 ± 4.0 mV. The presence of two protein G domains seems not to cause any significant change in the zeta potential value of the wild type protein, which is in accordance with a previous study reporting that protein G immobilized polymersomes have a zeta potential close to this value, i.e., −17.0 ± 0.2 mV [30]. Evidently, rSbpA-GG has the same capability as wtSbpA to recrystallize on the surface of emulsomes, and the entrapped curcumin has no significant influence on the S-layer recrystallization process. One may therefore speculate that the ability of the fusion protein rSbpA-GG to modify the surface of the emulsomes is independent of any loaded drug in low enough concentrations. Increasing the curcumin concentration was, however, found to affect the S-layer self-assembly; no recrystallization of rSbpA-GG was observed at a curcumin concentration of 110 μg mL^−1^.

### 3.7 Antibody binding characteristics of rSbpA-GG coated emulsomes

In previous studies, SbpA fusion proteins modified with some other functional moieties were recrystallized on positively charged liposomes [31], secondary cell wall polymer-coated solid supports [32,33] and microbeads [24]. Most importantly, the conformational structures as well as the characteristic features of the fused moieties were preserved. Likewise, immobilized on the surface, protein G domains must preserve their conformational 3D structure in order to present their inherent ability to recognize IgG. The S-layer rSbpA-GG attaches to the phospholipid surface via its N-terminus and is therefore expected to orient the two C-terminally fused protein G domains toward the outer face. This kind of C-terminus exposed orientation would contribute to prevention of possible nonspecific hydrophobic interactions between protein G and the lipid surface, thereby enabling the protein G domains to maintain their tertiary conformation. The IgG is therefore expected to bind to the modified emulsomes via its antibody tail (Fc) region, leaving the Fab portion available for antigen binding. The accuracy of this assertion was investigated as described below.

**Fig. 4:**
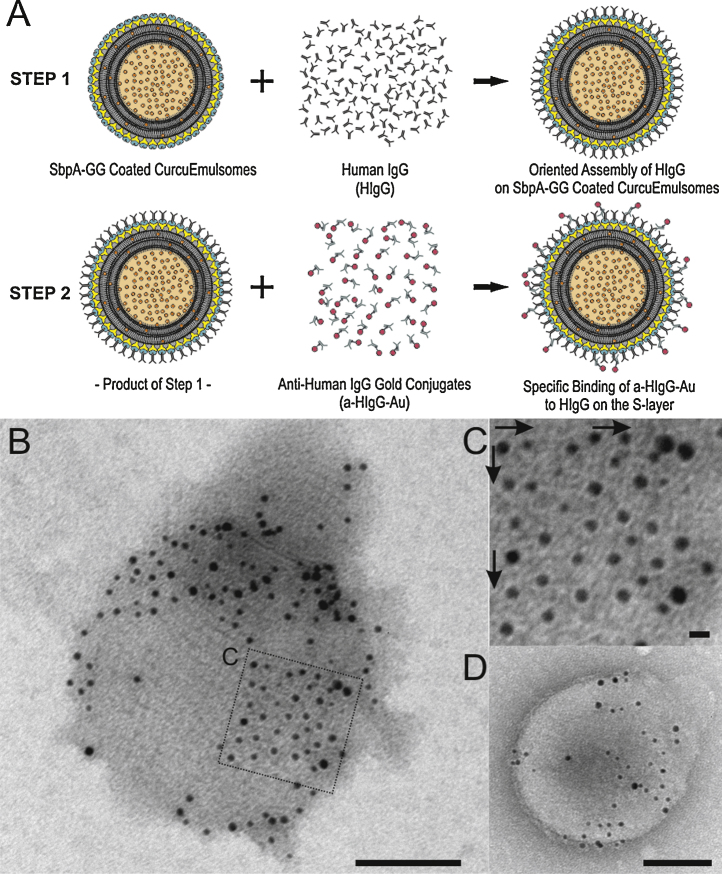
(A) Schematic drawing illustrating two-step indirect approach verifying the antibody binding to rSbpA-GG coated emulsomes. First, HIgG molecules are recognized by the rSbpA-GG coated CurcuEmulsomes and bind to the lattice. The α-HIgG-Au conjugates then interact with the bound HIgG. (B) A TEM image of an rSbpA-GG coated emulsome upon which HIgG and α-HIgG-Au conjugates were bound specifically. The scale bar corresponds to 100 nm. (C) Inset image, where the arrows indicate that HIgG binding follows the p4 symmetry on the S-layer lattice. The bar corresponds to 10 nm. (D) A TEM image of an rSbpA coated emulsome upon which HIgG and/or α-HIgG-Au conjugates were bound nonspecifically. The scale bar corresponds to 100 nm.

Anti-human IgG gold conjugates (α-HIgG-Au) were used to verify the antibody binding properties of the S-layer coated emulsomes. First, rSbpA-GG coated emulsomes were incubated with HIgG. The resulting product was then incubated with α-HIgG-Au which bound to HIgG (Fig. 4A) and enabled the detection of binding via TEM (Fig. 4B). The main advantages of this approach are that it confirms the binding while also indicating the locations of single binding sites and verifying the C-terminus oriented accessibility of protein G molecules on the lattice. The bound Au conjugates in several regions several regions can be seen to follow the same square symmetry of the underlying S-layer (Fig. 4C, arrows).

For a negative control, the same procedure was repeated with rSbpA coated emulsomes lacking the protein G domains. Some nonspecific binding was observed (Fig. 4D) and attributed to the affinity of S-layer protein for gold [34]. The relatively low binding of α-HIgG-Au on SbpA coated emulsomes indicated that the interaction between the S-layer and gold is weak compared to the strong interaction between HIgG and α-HIgG-Au. The specific IgG recognition of rSbpA-GG coated emulsomes was confirmed using an additional approach (Supplementary data Fig. 2), in which the interaction between the α-HIgG-Au and HIgG-FITC coated emulsomes was visualized under a confocal laser scanning microscope (Supplementary data Fig. 3).

## 4 Discussion

On the positively charged outermost shell of the emulsomes and CurcuEmulsomes, both wtSbpA and rSbpA-GG reassemble to the same coherent crystalline lattice with square symmetry (Fig. 3). The recrystallization of rSbpA-GG on the surface of CurcuEmulsomes causes a shift in the zeta potential of the nanocarrier from 29.8 ± 2.1 mV to −22.7 ± 2.1 mV (Table 1), thereby confirming the nanocarriers are completely covered with the S-layer of rSbpA-GG. Clearly, these findings provided evidence that CurcuEmulsomes can be coated with rSbpA-GG and that the presence of curcumin inside the nanocarrier at the concentration used here (i.e., 30 μg mL^−1^) had no influence on the S-layer recrystallization characteristics. It is important to emphasize that this curcumin concentration is sufficiently high to enable its medical use (i.e., IC_50_: 2–40 μg mL^−1^ [35]).

As recently reported, it is possible to increase the curcumin content of CurcuEmulsomes up to 110 μg mL^−1^ [19]. At this concentration, the curcumin was found to affect the self-assembly of the S-layer proteins, preventing the recrystallization of rSbpA-GG. This may be due to nonspecific absorption of curcumin on the surface of the emulsome. As a result, the recrystallization process of the S-layer protein may be disturbed. On the other hand, it is also possible that incorporation of curcumin in very high amounts may influence the stiffness or roughness of the outermost phospholipid bilayer of the nanocarrier, factors which can affect the recrystallization [36]. A full understanding and clarification merits a detailed further study, which is out of the scope of this work.

The relative IgG affinities of rSbpA-GG, SbpA and SbpA-ZZ were qualitatively evaluated by monitoring the binding of POX labeled goat IgG. The results obtained clearly showed that goat IgG binds strongly only to plates coated with rSbpA-GG; the rSbpA-ZZ with its protein A domains had a very low affinity for goat IgG, and rSbpA displayed almost no binding (Fig. 2). These data indicated that rSbpA-GG possesses strong affinity toward IgG which is conferred by the two fused protein G domains. SbpA lacking the protein G moieties does not have any affinity for IgG. In addition, our data verified once again that – unlike protein G – protein A shows lower binding to IgGs from several animal species such as goat [37].

Following the recrystallization of rSbpA-GG on emulsomes, HIgG binding affinity of tailored emulsomes was examined by an indirect approach (Fig. 4A) in which HIgG binding on the S-layer was demonstrated by the subsequent coupling of α-HIgG-Au conjugates. This approach provided not only evidence for binding, but also showed that HIgG binding to the S-layer follows the p4 lattice symmetry (Fig. 4B). Confocal laser scanning microscopy analysis with FITC-labeled HIgG provided further evidence that HIgG specifically binds to the rSbpA-GG lattice on the emulsomes, and hence, rSbpA-GG coated emulsomes are functional in terms of specific IgG recognition (Supplementary data, Fig. 3). Again, lacking the functional protein G domains, SbpA coated CurcuEmulsomes do not display any specific IgG binding.

TEM analysis revealed that not all presented protein G domains within the lattice were occupied by α-HIgG-Au conjugates. This may be attributed to the fact that either (i) α-HIgG-Au's could not bind to all free HIgG's immobilized on the S-layer, or (ii) not all binding sites on the S-layer, i.e., protein G domains, were saturated with HIgG. Besides these kinetic parameters, interactions and collisions occurring between emulsomes may also limit antibody binding, as suggested by a previous study [10]. This argument could explain why the α-HIgG-Au-occupied regions are present as patches instead of the α-HIgG-Au being distributed homogeneously on the S-layer lattice (Fig. 4B).

**Fig. 5:**
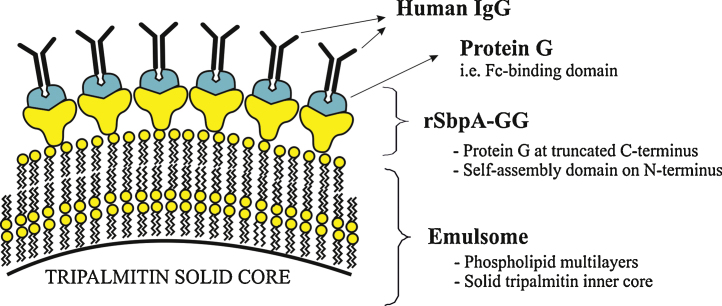
Schematic drawing illustrating the immobilization of HIgG on rSbpA-GG coated emulsomes. Following the rSbpA-GG recrystallization on the phospholipid multilayer surface of emulsomes, HIgG binds in a regular manner via the F_C_ region to protein G domains.

As alternative to our genetic approach, HIgG could be covalently (i.e., chemically) linked to the S-layer lattice as previously reported [38–40]. However, the present approach using site-mutagenesis benefits from the specific interactions that make the antibody binding (F_ab_) regions to become accessible for antigen binding (Fig. 5). This inherent control over orientation of HIgG may further contribute to direct the CurcuEmulsomes toward IgG specific cells, in particular inflammatory and cancer cells [11], a topic that will be a focus of our forthcoming studies.

In nanomedicine, particular care has to be taken with the particles’ surfaces to avoid innate immune system recognition and to secure sufficiently long circulation half-lives for the agents to reach their targets [41]. As the most common approach, surface-bound biocompatible polyethylene glycol (PEG) allows the formation of a hydrated steric barrier that decreases nanocarrier interaction with blood-borne components. This causes an increased blood circulation time, decreased spleen and liver capture, and improved tumor uptake [22]. The PEG cushion reduces the adhesion of opsonins present in the blood serum on nanoparticles [42], and the immunological response is reduced [43]. The S-layer proteins described here may provide an alternate approach for surface modification. The S-layer protein SbpA was recently shown to form (at basic pH) smooth, cytophobic patterns that eliminate the adsorption of human plasma proteins [44], as well as the adhesion of cells (e.g., HepG2) [45]. The SbpA coating may therefore minimize the adhesion of opsonins and enhance the circulation time of the modified emulsomes. These anti-fouling characteristics of the SbpA layer are expected to benefit CurcuEmulsomes in vivo, potentially improving the circulation time. The verification of this effect requires further investigations in vivo, which is foreseen in our forthcoming studies.

## 5 Conclusions

The present study introduces CurcuEmulsomes coated with rSbpA-GG as a nanoparticulate DDS that mimics a viral envelope. The S-layer fusion protein was shown to form a uniform monomolecular lattice on the surface of the emulsomes and the CurcuEmulsomes, altering the surface characteristics of the lipid-based nanocarrier and bestowing IgG binding functionality on the nanocarrier. Entrapped curcumin at a concentration of 30 μg mL^−1^ did not influence the self-assembly characteristics of the S-layer protein. This study indicates that S-layer fusion technology is a highly effective approach for immobilization of foreign proteins such as protein G domains on emulsomes. The distinct advantage of using S-layer proteins is that they can be recrystallized in an oriented fashion on a variety of supports including spherical surfaces covered by phospholipids [46]. Previous studies have also shown that mixtures of native S-layer proteins [47] and S-layer fusion proteins incorporating different functional domains [46] assemble into coherent monomolecular layers on different surfaces including liposomes [48]. Moreover, the predetermined orientation of the proteins in the S-layer lattice ensures that the functional regions of the foreign protein (such as F_ab_ regions) will be accessible for functions such as antigen binding. The high affinity of HIgG toward rSbpA-GG on the surface of CurcuEmulsomes underlines the potential of the proposed system for in vivo drug delivery. Such multi-faceted and versatile nanocarriers and drug delivery systems promise a substantial increase in the efficacy of diagnostic and therapeutic applications in pharmaceutical sciences.

## Figures and Tables

**Table 1:** Average values for zeta potential of emulsomes and CurcuEmulsomes before and after being coated with rSbpA-GG fusion protein.

	Zeta potential before rSbpA-GG coating (mV)^a^	Zeta potential after rSbpA-GG coating (mV)
Emulsome^b^	32.4 ± 5.9 mV	−19.5 ± 3.7 mV
CurcuEmulsome^c^	29.8 ± 2.1 mV	−22.7 ± 3.7 mV

a Data were recorded at the day of recrystallization.

b More than 10 separate samples with average conductivity of 0.158 ± 0.011 mS cm^−1^.

c 10 separate samples with average conductivity of 0.175 ± 0.003 mS cm^−1^. Standard deviations given by the ±values correspond to the average standard deviation of all measurements, where *n* ≥ 3.
