# Supplementary material for: S-layer fusion protein as a tool functionalizing emulsomes and CurcuEmulsomes for antibody binding and targeting
Source: Colloids Surf B Biointerfaces. 2015 Apr 1;128:132–9. doi: 10.1016/j.colsurfb.2015.01.055 (PMC4406452; doi:10.1016/j.colsurfb.2015.01.055)
Supplement: Supplementary file 1 [file mmc1.docx]

**S‑layer Fusion Protein rSbpA‑GG as a Tool functionalizing Emulsomes and CurcuEmulsomes for Antibody Binding and Targeting**

*Mehmet H. Ucisik^a,b,^*, Seta Küpcü^b^, Andreas Breitwieser^c^, Nicola Gelbmann^d^, Bernhard Schuster^b^, Uwe B. Sleytr^e^*

# **Supplementary Data**

**Supplementary Methods**

Preparation of rSbpA‑GG self-assembly for TEM analysis: Monomeric and/or oligomeric rSbpA‑GG solutions were recrystallized on poly‑L‑lysine (PLL) coated copper grids as described previously by Pum et al. (1989).[[1](#_ENREF_1)] In brief, formvar- and carbon coated grids were glow discharged and floated on a drop of a poly-L-lysine (Sigma P2636) solution (0.1% in Milli-Q water) for 10 min. After excess polyL-lysine was removed, the grids were incubated with the self-assembly suspension for 10 min to 2 h at 20°C and subsequently used for negative staining.

Preparation of HIgG‑FITC conjugates: 7 mg HIgG was dissolved in 950 µl NaHCO_3_/Na_2_CO_3_ buffer (pH: 9.5). After the addition of 50 µl FITC solution (10 mg ml^-1^ in DMSO; FITC from Sigma Aldrich, Germany) the mixture was incubated 1 hour in the dark at room temperature, with rotation. Unreacted FITC was removed with a PD10 desalting (or gel filtration) column. Before use, PD10 column was prewashed thoroughly with 20 ml PBS.

Affinity assay with HIgG‑FITC conjugates: 20 µl of rSbpA-GG coated emulsome solution (corresponding 10 µg DPPC content), 30 µl of PBS and 50 µl of 1 mg ml^-1^ HIgG‑FITC solution were mixed and incubated for 15 min at 20^o^C and 800 rpm in eppendorf mixer. Then it was centrifuged at 14,100 g for 3 min. The supernatant was discarded; the pellet was dissolved in 20 µl of 1 mg ml^-1^ HIgG‑FITC solution and the incubation was repeated for 15 min. After 3 min centrifugation, pellet was dissolved in 20 µl PBS and the sample was kept at 4^o^C until analysis with the CLSM. Emulsomes were priory loaded with Sudan III to make the identification of emulsomes possible.[[2](#_ENREF_2)] Sudan III was preferred instead of curcumin due to its higher fluorescence signal.

Atomic Force Microscopy (AFM): Silicon wafers with native oxide layer were cut into pieces 1x1 cm^2^ in size. The wafers were O_2_ plasma-treated (Gala Instruments, Germany) as described.[[3](#_ENREF_3)] The hydrophilic silicon supports were used for protein recrystallization immediately after plasma treatment. The rSbpA‑GG protein solution used for recrystallization experiments was prepared by dilution of the supernatant of the protein extract at a ratio of 1:10 in 0.5 mm tris(hydroxymethyl)aminomethane/HCl buffer (pH 9) containing 10 mM CaCl_2_. The final concentration of the protein monomers was ≤ 1 mg ml^-1^.

All experiments were performed in contact mode in water or aqueous solutions of NaCl (0.1 Μ) at room temperature, by using a multimode atomic force microscope and a Nanoscope V controller (Veeco Instruments, Santa Barbara, CA) equipped with E-Scanner as described.[[4](#_ENREF_4)] Silicon nitride cantilevers with a nominal spring constant of 0.1 Nm^-1^ (NP, NP-S, Veeco Instruments) were used for imaging. The scanning force was adjusted to be below 0.5 nN to minimize tip-induced damage of the sample.

Confocal laser scanning microscopy (CLSM): CLSM analyses were performed with a Leica TCS SP5 II system (Leica, Wetzlar, Germany). Images were taken with a 63x/1.20 water immersion objective. Emulsomes filled with Sudan III were excited at wavelength λ = 561 nm and detected in the range of 570 nm – 650 nm. Images were acquired and processed with Leica LAS AF software.

**Supplementary Results**

Self-assembly properties of rSbpA-GG

For evaluating the self‑assembly properties of rSbpA-GG, purified proteins were dialyzed against 10 mM CaCl_2_ for 18 h at 4^o^C. As shown by TEM images of negatively stained preparations, rSbpA-GG reassembled into flat sheets, which clearly exhibited the square (p4) lattice structure of wildtype SbpA (Supplementary Figure 1‑A). The self‑assembly characteristics of the rSbpA-GG were also verified on planar silicon wafer via AFM studies (Supplementary Figure 1-B).


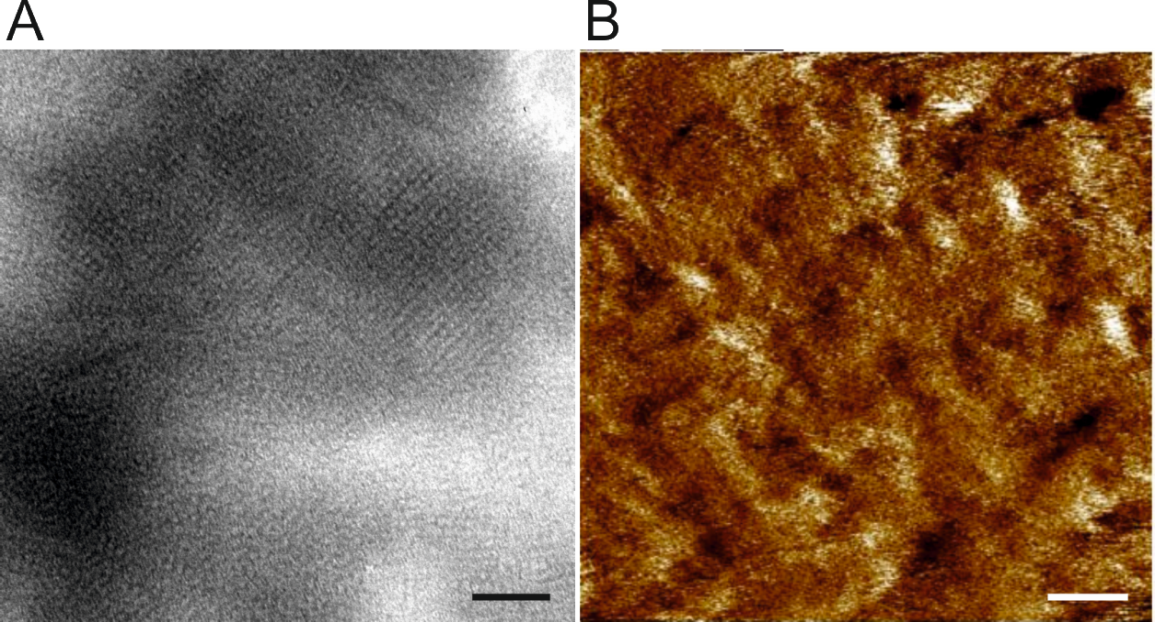


**Supplementary Data Figure 1.** (A) Electron micrograph of the rSbpA-GG lattice recrystalized on Poly-L-lysine coated copper grid; (B) AFM image of rSbpA‑GG layer assembled on silicon wafer. Bars correspond to 100 nm.

Fluorescent detection of specific IgG binding properties of rSbpA‑GG

In this approach, HIgG-FITC (fluorescein isothiocyanate) conjugates were synthesized for detection of the IgG affinity of rSbpA‑GG coated emulsomes by means of CLSM. This one‑step approach (Supplementary Figure 2) comprises incubation of rSbpA‑GG coated emulsomes with the prepared HIgG‑FITC conjugates, where the latter are expected to specifically bind with their F_c_ domain to the free protein G molecules exposed by the S‑layer; thereby covering the S‑layer coated nanocarrier.


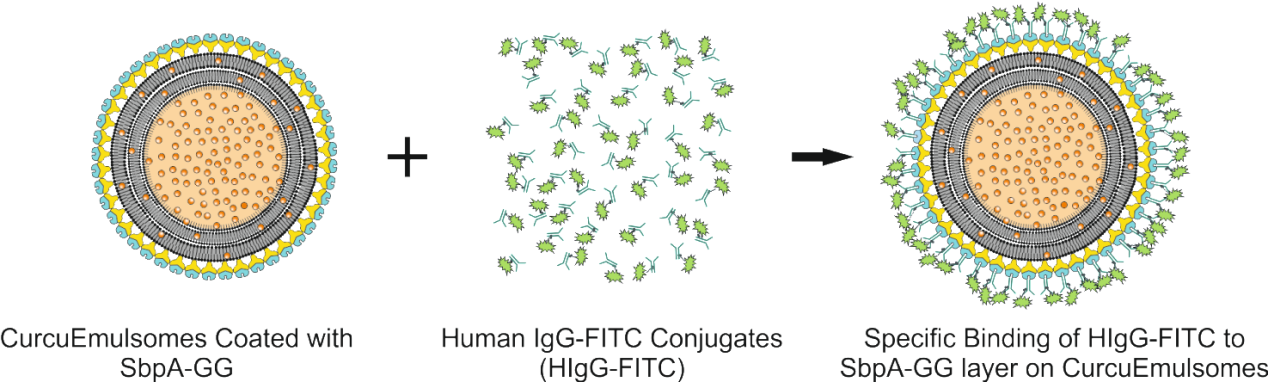


Supplementary Data Figure 2. Schematic drawing illustrates the experimental procedure for fluorescent detection of IgG‑binding.

The final product of the illustrated process was analysed by CLSM, which indeed verified the binding of HIgG-FITC to rSbpA‑GG (Supplementary Figure 3). In the image, the green shell‑like regions represent the human IgG‑FITC molecules attached on the protein G domains of the rSbpA‑GG lattice, whereas the red sphere inside denotes the dye Sudan III loaded emulsomes.


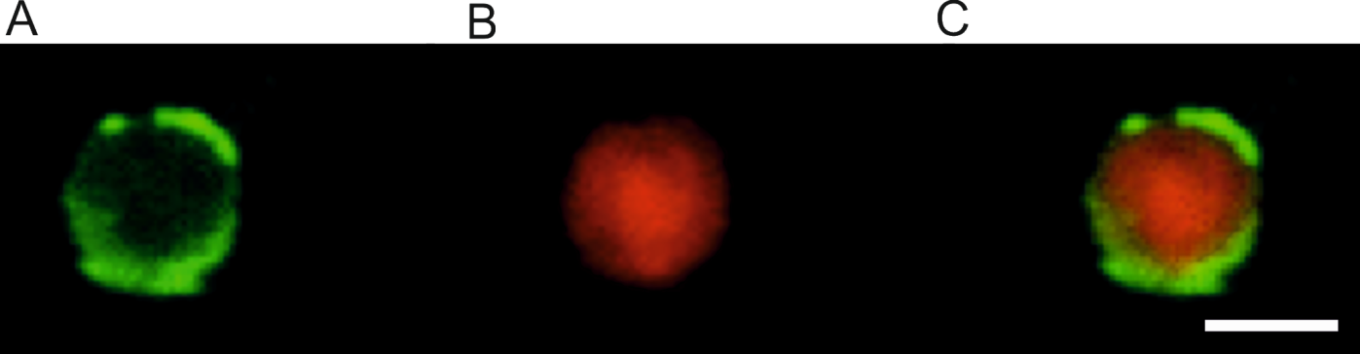


Supplementary Data Figure 3. CLSM image of rSbpA-GG coated emulsomes interacting with HIgG-FITC conjugates. (A) Surface-bound HIgG‑FITC molecules form a shell around the emulsome; (B) Sudan III loaded in the solid inner fat core is seen as a red sphere; (C) Overlay image of A and B. Sample was excited at a wavelength of 561 nm and emission was detected in the range of 570 nm – 650 nm. Bar represents 2 µm.

[1] D. Pum, M. Sára and U.B. Sleytr, J Bacteriol, 171 (1989) 5296-5303.

[2] M.H. Ucisik, S. Küpcü, M. Debreczeny, B. Schuster and U.B. Sleytr, Small, 9 (2013) 2895-2904.

[3] J.L. Toca-Herrera, S. Moreno-Flores, J. Friedmann, D. Pum and U.B. Sleytr, Microscopy Research and Technique, 65 (2004) 226-234.

[4] S. Moreno-Flores, A. Kasry, H.-J. Butt, C. Vavilala, M. Schmittel, D. Pum, U.B. Sleytr and J.L. Toca-Herrera, Angewandte Chemie, 120 (2008) 4785-4788.
